# Consensus Head Acceleration Measurement Practices (CHAMP): Laboratory Validation of Wearable Head Kinematic Devices

**DOI:** 10.1007/s10439-022-03066-0

**Published:** 2022-09-14

**Authors:** Lee Gabler, Declan Patton, Mark Begonia, Ray Daniel, Ahmad Rezaei, Colin Huber, Gunter Siegmund, Tyler Rooks, Lyndia Wu

**Affiliations:** 1 Biomechanics Consulting and Research, LLC, Charlottesville, VA USA; 2 Center for Injury Research and Prevention, Children’s Hospital of Philadelphia, Philadelphia, PA USA; 3 Department of Biomedical Engineering and Mechanics, Virginia Tech, Blacksburg, VA USA; 4 United States Army Aeromedical Research Laboratory, Fort Rucker, AL USA; 5 Department of Mechanical Engineering, The University of British Columbia, Vancouver, BC Canada; 6 Department of Bioengineering, University of Pennsylvania, Philadelphia, PA USA; 7 MEA Forensic Engineers & Scientists, Richmond, BC Canada; 8 School of Kinesiology, University of British Columbia, Vancouver, BC Canada

**Keywords:** Accuracy, Best practices, Head impact kinematics, Recommendations, Validation

## Abstract

Wearable devices are increasingly used to measure real-world head impacts and study brain injury mechanisms. These devices must undergo validation testing to ensure they provide reliable and accurate information for head impact sensing, and controlled laboratory testing should be the first step of validation. Past validation studies have applied varying methodologies, and some devices have been deployed for on-field use without validation. This paper presents best practices recommendations for validating wearable head kinematic devices in the laboratory, with the goal of standardizing validation test methods and data reporting. Key considerations, recommended approaches, and specific considerations were developed for four main aspects of laboratory validation, including surrogate selection, test conditions, data collection, and data analysis. Recommendations were generated by a group with expertise in head kinematic sensing and laboratory validation methods and reviewed by a larger group to achieve consensus on best practices. We recommend that these best practices are followed by manufacturers, users, and reviewers to conduct and/or review laboratory validation of wearable devices, which is a minimum initial step prior to on-field validation and deployment. We anticipate that the best practices recommendations will lead to more rigorous validation of wearable head kinematic devices and higher accuracy in head impact data, which can subsequently advance brain injury research and management.

## Summary Statements

This work was part of the Consensus Head Acceleration Measurement Practices (CHAMP) project. The objective of CHAMP was to develop consensus best practices for the gathering, reporting, and analysis of head acceleration measurement data in sport. Subject matter experts were recruited to draft a series of papers on various aspects of the issue. As described in detail in a companion paper,^3^ each team drafted a paper and several summary statements ahead of the CHAMP Consensus Conference, held on March 24–25, 2022, at the Children’s Hospital of Philadelphia. The following summary statements were discussed, revised as necessary, and ultimately approved by more than 80% of the vote at the conference:

1. A wearable device that measures head acceleration must be independently validated for its intended application through controlled laboratory testing, and the laboratory should simulate the real-world loading environment in which the device will be used.
2. Laboratory testing of wearable devices should use a validated biofidelic anthropomorphic test device (ATD) headform combined with a repeatable and reproducible test setup that enables testing across multiple levels of magnitude, duration, and direction that simulate on-field linear and angular head kinematics relevant to the setting of study.
3. Reference sensor setup and validation metric selection depend on the intended application of the wearable device, which can vary on four main levels: impact counting, impact magnitude, impact direction, and the time-history measurement of six-degree-of-freedom (6DOF) head kinematics.
4. If a wearable device is designed to measure and report metrics derived from head kinematics, ground truth measurements must be collected with an ATD-embedded laboratory-grade reference sensor system. If a wearable device is designed to count impacts only, a reduced reference setup enabling verification of impact events may be applied.
5. Processed data from the wearable device must be compared with ground truth measurements using validation metrics and statistical methods that enable complete, unbiased, and application-relevant assessment of accuracy and uncertainty.

We recommend that wearable devices undergo laboratory validation according to the CHAMP best practices outlined in the current paper, and this should be stated within the corresponding validation documentation or publication.

## Introduction

Wearable devices have the potential to measure real-world head impacts occurring in sport.^21,60,65,70^ An increased interest in head impact monitoring coupled with advancements in sensing technology has led to the development of various devices,^65,90,94^ that include kinematic sensors mounted in helmets,^2,20,71^ headbands,^35^ skin patches,^94^ earpieces,^63,73^ and mouthpieces.^7,16,25,56^ These devices often leverage small, low-cost, low-power sensor technologies, with the most incorporated sensors being microelectromechanical system (MEMS) accelerometers to measure linear acceleration and gyroscopes to measure angular velocity. While wearable devices make it possible for large-scale research deployment and consumer adoption, they are often limited in accuracy due to limitations in low-cost sensor capabilities, imperfect head-device coupling, and the complexity of real-world impact conditions. Therefore, a wearable device must be validated, i.e., proven to be accurate, for its intended application by undergoing controlled laboratory testing.

Despite the wide range of laboratory test setups published, little effort has been made to establish standard approaches to validate wearable devices in the laboratory.^42^ Wearable devices are applied in research or offered to consumers as turnkey systems with incomplete or irrelevant validation testing, or without any published validation information. Ideally, the laboratory would simulate the loading environment in which the device will be used. Human surrogates for device mounting should be selected based on their biofidelity and ability to mimic real-world coupling between the device and head. Test conditions should be repeatable, reproducible, and chosen according to how well they represent the on-field impact scenarios and mechanics. Data from the device should be collected, processed, and analyzed in an unbiased manner relative to ground truth measurements from reference sensors. These aspects are important to consider when assessing wearable device accuracy in the laboratory.

The proliferation of wearable devices facilitate access to valuable human participant data that may not otherwise be accessible. However, high variability in validation methods lead to inconsistent and often incomplete device evaluation. In addition, users of these systems may be unaware of the limitations when using a specific device in a particular application.^65^ Insufficient understanding of the limitations of a device may result in incorrect conclusions that could confound the collective knowledge of a topic and potentially impede safety advancements. Therefore, establishing best practices for validating the accuracy of wearable devices in a laboratory setting would benefit both developers and users.^29^

This paper summarizes best practices developed by an expert consensus group for validating the accuracy of wearable devices for measuring head impacts in the laboratory. Laboratory validation should be an initial step in examining the accuracy of a wearable device, and further on-field validation is likely required to confirm device accuracy in an on-field setting (best practices for on-field validation are included in a companion paper).^44^ Accuracy is defined as the degree to which measurements made by a wearable device match those from ground truth (reference) sensors. Repeatability is defined as the degree of variation in the measurement.^12,36^ Head impacts are defined as a subset of head acceleration events (HAEs) that result from forces applied directly to the head or protective headgear. Wearable devices may also capture inertial loading of the head (i.e., from body contact without direct head contact); however, the best practices presented in this paper do not focus on validating wearable sensors under these loading conditions. Aspects of wearable device validation covered in this paper focus on impact counting and other features derived from kinematic measurements: impact magnitude, impact direction, and the complete time-history measurement of six-degree-of-freedom (6DOF) head kinematics. Recommendations and best practices for laboratory validation are divided into four separate topics to be discussed in detail in the following sections: surrogate selection, test conditions, data collection, and data analysis. Each section is organized into key considerations that address fundamental factors that influence wearable device accuracy, recommended approaches for validation methods, and specific considerations with examples of best practices approaches.

## Surrogate Selection

When selecting a test surrogate, the overall goal is to choose a model of the head that provides the most biofidelic platform for mounting the wearable device and reference sensor for impact testing.

### Key Considerations

**Figure 1:**
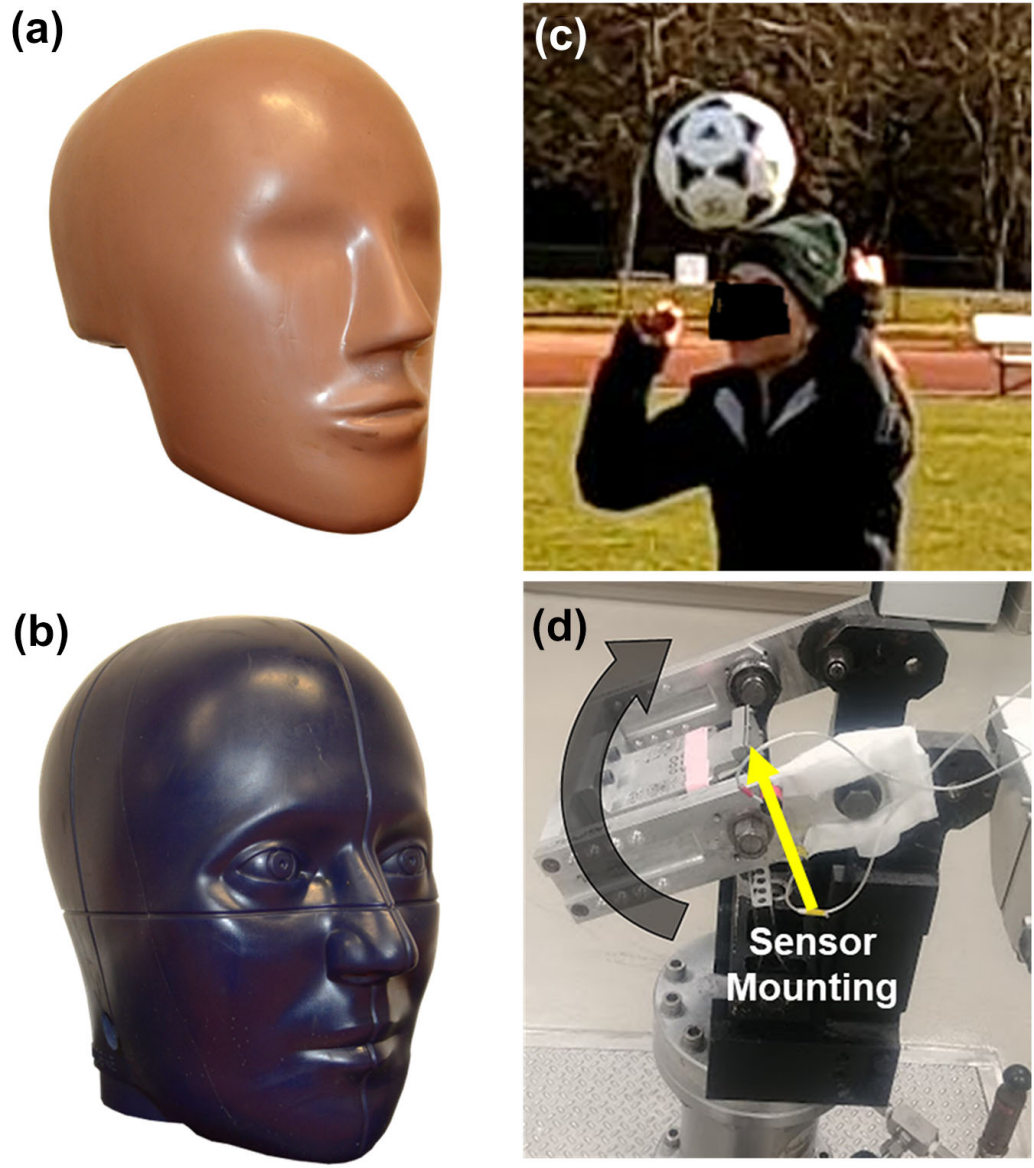
Examples of test surrogates that have been used to assess the performance of wearable devices in the laboratory. (a) Hybrid III and (b) NOCSAE 50th percentile adult male ATD headforms, (c) a volunteer heading a soccer ball, and (D) an example of a non-biofidelic test device (the HYGE Rotational Motion Device).^35^

Because the objective of the laboratory tests is to validate a wearable device for head impacts, a physical surrogate model of the human head is needed. How well this model represents the human head, i.e., its biofidelity, in the context of head impacts is a key consideration for surrogate choice. Biofidelity can include overall geometry, inertial properties, tissue mechanical properties (e.g., soft tissue deformation behavior, skin or hair friction), and specific anatomical features (e.g., dentition). Head to wearable device coupling is also an important consideration and is dependent on the specific anatomical features and tissue mechanics. An important practical consideration is the ability of the surrogate to enable ground-truth head impact measurements as a reference to evaluate device performance. Additional considerations include the repeatability of the surrogate, the maximum possible impact severity, cost, and ethics. Four main types of surrogates have been used to evaluate wearable device accuracy in the laboratory: non-biofidelic test devices, anthropomorphic test devices (ATDs), post-mortem human subjects (PMHS), and human volunteers (Fig. 1). For each surrogate, the advantages and limitations are weighed against the key considerations (Table 1).

**Table 1:** Key considerations for surrogate selection.

Surrogate	Type	Biofidelic geometry and inertial properties	Biofidelic tissue mechanics	Reference measurement	Repeatability	Max severity	Cost	Ethical considerations
Non-biofidelic test devices	Mechanical	No	No	Yes	Excellent	High	Low	No
ATDs	Mechanical	Limited	Limited	Yes	Good	High	Moderate	No
PMHS	Biological	Yes	Yes	Limited	Limited	High	High	Yes
Human volunteers	Biological	Yes	Yes	Limited	Limited	Minimal	Low	Yes

### Recommended Approach

ATD headforms are designed to represent the geometric and inertial properties of the human head, include built-in reference sensor mounting options, and offer good repeatability and reproducibility in non-destructive testing over a range of impact severities.^59^ We recommend that laboratory testing of wearable devices use a standard ATD headform validated to support the intended application, because standardized commercial options of ATD headforms are available to users for achieving a repeatable and reproducible outcome. While biological or custom surrogates may also produce similar outcomes, those conducting the validation need to have more specialized experience working with the corresponding surrogates. When choosing an ATD, consider the match between conditions of the ATD validation and the intended application. Depending on the objectives of the study and the type of device, use of a modified or validated custom test surrogate may be appropriate. This approach may be needed in situations where a standard headform cannot accommodate the mounting requirements (see Data Collection section for more details). Because sensor-head coupling is a critical factor in accurately measuring head kinematics,^94^ it is important to replicate biofidelic device mounting (e.g., instrumented mouthguards need to be mounted on a surrogate’s dentition, skin devices need to be mounted on the surrogate’s skin material, and ear-mounted devices within the ear). When designing or choosing a modified or custom surrogate, consider potential new sources of error that could be introduced when using the surrogate, and the validity of the surrogate in the intended test conditions.^80^

### Specific Considerations

Validated, standard ATD headforms include, for example, the Hybrid III headform,^22^ National Operating Committee on Standards for Athletic Equipment’s (NOCSAE) headform,^33^ EuroSID-2 side impact dummy,^15^ and ADAM manikin.^6^ The two most common surrogates used for testing wearable devices are the Hybrid III and NOCSAE 50th percentile adult male headforms (Fig. 1). The Hybrid III head consists of an aluminum skull with removable vinyl nitrile skin and was developed and validated for frontal crash tests.^22,55^ The Hybrid III headform skin includes simplified facial features and can be connected to a neckform, however, it is missing a mandible and a nape. The NOCSAE headform was developed to evaluate the safety of American football helmets in drop tests and consists of a gel‐filled cavity encased by a urethane shell with anatomical features including a nape, chin, and cheeks.^33^ Prior studies have observed differences in head kinematic response between these two ATDs.^41^ While both headforms are constructed with deformable materials on the exterior, the materials may not simulate soft tissue behavior during impacts for devices mounted to the soft tissue or scalp (e.g., skin patches or helmet-mounted devices).^11,94^

Standard headforms have been modified in past studies to test specific types of devices. The Hybrid III headform was modified by carving a hole into the skin to accommodate an in-ear mounted device.^76^ The NOCSAE headform has been modified to accommodate instrumented mouthpieces by carving an opening in the mouth.^32,56,69^ A modified version of the Hybrid III head called the mandibular load-sensing headform (MLSH) was validated against an unmodified Hybrid III headform for the purpose of evaluating the accuracy of instrumented mouthpieces and helmet-mounted devices.^17,80^ To validate an instrumented mouthguard, a custom-built headform was developed to accommodate biofidelic dentition (X2 headform).^16^ Although the X2 headform has the size, mass, and center-of-gravity (CG) of the 50th percentile male human head, no impact biofidelity and repeatability validation testing was performed. Modified headforms can simulate biofidelic mounting of wearable devices, which can be crucial when evaluating accuracy; however, rigorous validation is required. For example, the presence of the mandible is an important factor when testing instrumented mouthpieces.^23^ Sensor accuracy in tests with an unconstrained mandible was severely diminished (up to 80% error) when compared to conditions in which the mandible was fixed or completely removed.^25,45^

A neckform can be added to a headform to simulate head-neck response to impact and can often enable higher rotational head kinematics. Examples of neckforms that have been validated under frontal impact in automotive applications include the Hybrid III neck^22^ and THOR neck.^78^ Most studies use the Hybrid III neck given its availability, durability, and extensive use in impact testing despite having a stiffer response when compared to initially-relaxed humans at lower severities,^82^ when in compression,^74^ or torsion.^64^ The Hybrid III neck is comprised of rubber discs and aluminum plates which are intended to simulate human vertebrae.^22^ The NOCSAE headform can be modified to mount on the Hybrid III neck^9^; however, the commercially-available neck adaptor is recommended due to concerns regarding durability of the head-neck interface at high speed impacts.^30^ While the Hybrid III neck has good repeatability,^50^ temperature effects and recovery intervals between tests have been found to influence neck stiffness and should be controlled. For example, the compressive stiffness of the Hybrid III neck was 15% lower at 37.5 °C compared to 25 °C, whereas the stiffness more than doubled at 0 °C.^77^

Despite the advantages of ATDs, certain biofidelity features can be difficult to replicate in ATDs, such as soft tissue mechanics, which could be key factors in the performance of devices mounted directly or indirectly on soft tissue. In a PMHS study, a skin-mounted device overestimated peak linear acceleration and peak angular acceleration by 64 ± 41% and 370 ± 456%, respectively,^79^ whereas similar tests in ATDs showed much lower error levels (up to 24% in linear acceleration and up to 57% in angular acceleration).^88^ PMHS are seldom used to test wearable devices due to a number of challenges; however, they provide a biofidelic platform that can be used to answer specific questions about sensor accuracy that cannot be accomplished using other surrogate-types.^70^ PMHS have been used to study the interaction between the mandible and an instrumented mouthguard to examine structural characteristics of head impacts and determine bandwidth and sampling requirements for wearable devices.^45,93^ Despite the advantages of using PMHS, they are costly relative to tests involving ATDs, reference instrumentation cannot be easily placed, and require specialized facilities and expertise to handle. PMHS, like human subjects, have inherent variability, which limits repeatability. Furthermore, loading conditions for human volunteer studies are limited to minimal severity and making laboratory-grade reference measurements is challenging with volunteers.^94^

Non-biofidelic test devices offer a repeatable and low-cost solution for a basic evaluation of wearable device function. Examples include custom test fixtures mounted to drop towers, rotary devices, or shaker tables that are capable of generating uniaxial motions.^7,35^ They are useful in situations in which it is desirable to assess basic functionality and are a recommended first step in laboratory validation of wearable devices to isolate the intrinsic error of the sensing elements (e.g., MEMS accelerometers and gyroscopes) from other sources such as sensor-skull coupling.^35^ In some cases, only the sensing elements and supporting electronics are tested, whereas in others the complete wearable device is tested.^7^ Because the goal is usually to isolate the error of the sensor, it is important that the sensing elements be tightly coupled with a laboratory-grade reference sensor through the surrogate. While advantages of using non-biofidelic test devices may be realized during the developmental stage, the results from these tests cannot be used to infer the on-field validity of a wearable device.

## Test Conditions

The overall goal is to create repeatable laboratory test conditions that mimic, as closely as possible, the on-field environment that the wearable device will be exposed to in normal use.

### Key Considerations

**Figure 2:**
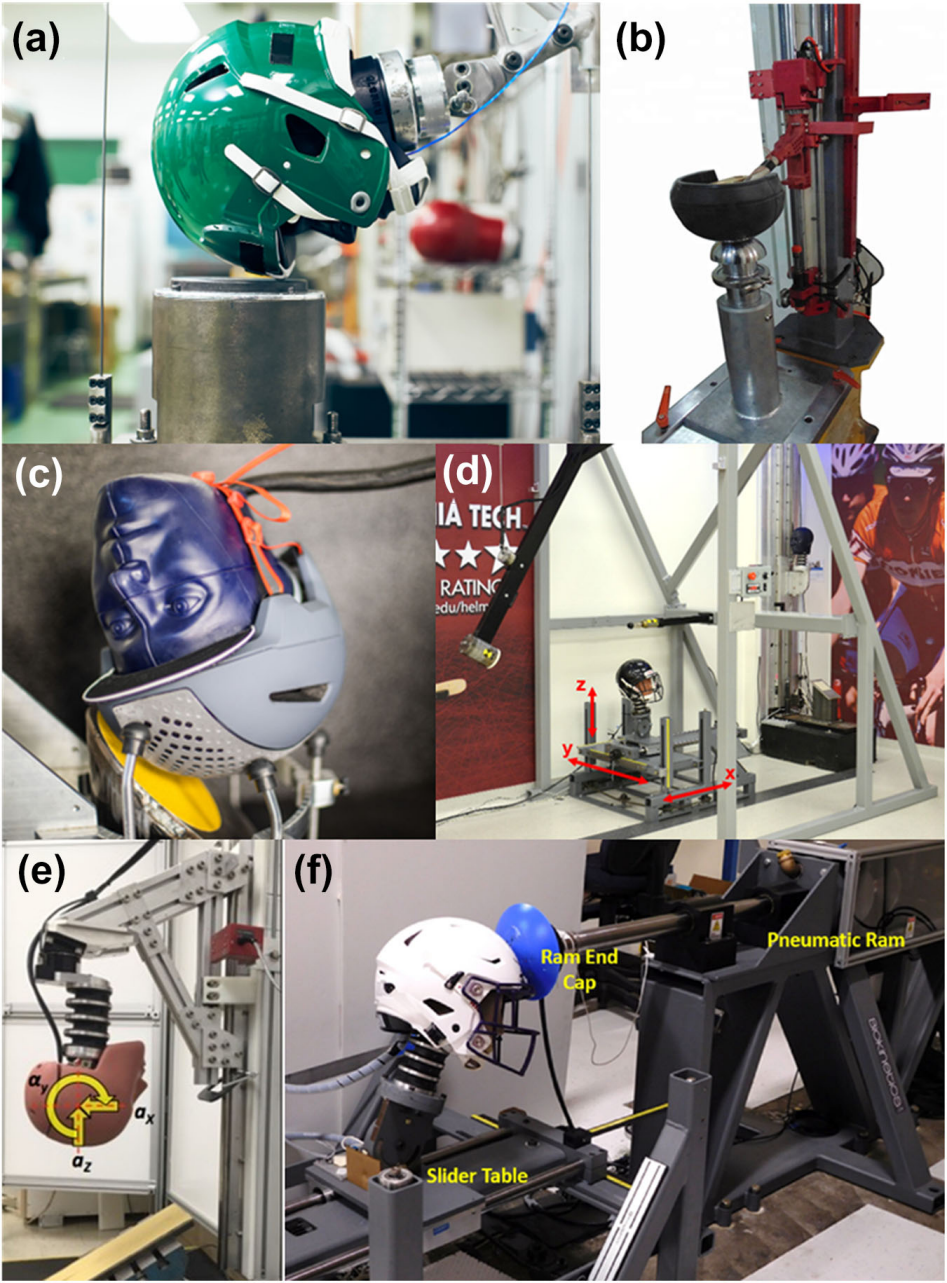
Examples of laboratory test setups that have been used to assess the performance of wearable devices. These include **A** a twin-wire drop tower with padded anvil, **B** a monorail drop tower with curved steel anvil, **C** a free drop onto an angled surface, **D** a pendulum impactor, **E** a complex drop test onto an angled surface, **F** and a linear impactor with curved impact surface

When selecting test conditions, it is important to consider how well the on-field impact conditions can be recreated in the laboratory. Thus, how closely the speed, location, and direction of the impact mimic the on-field conditions are key considerations. Typical head impacts associated with brain injury in sport occur due to direct high-energy impact between the head and other objects (e.g., helmet, body, and ground), and shown from limited field data, they involve short-duration, high linear and angular head acceleration.^23,31,66,72^ These conditions should be identified and replicated in the laboratory to cover the range of possible impact characteristics that the device could encounter. Additional considerations include the repeatability (intra-laboratory) and reproducibility (inter-laboratory) of the test conditions, the maximum possible impact severity, and cost. Several test setups have been used to evaluate wearable device performance in the laboratory (Fig. 2).

### Recommended Approach

Consider the target application, then select test setup and impact conditions that can replicate the magnitude, duration, and direction of the impacts measured on-field. Pneumatically or gravity driven impactors, used with slider tables mounted with biofidelic test surrogates enable a wide range of repeatable impact conditions for evaluating wearable device accuracy (Fig. 2). Select a range of impact speeds, and locations, in addition to surrogate orientation based on available on-field data.^5,90,91^ Select impactor surface and mass that represent the on-field conditions.^5^ Test conditions that generate known errors should not be excluded to artificially boost device performance. One key factor that affects device performance is how well it couples to the skull. Thus, on-field device coupling conditions should be simulated in laboratory testing. Tests in particular impact directions or locations and direct interference of wearable devices with other objects have also been shown to introduce substantial error.^45,73,81,92,94^ For example, impacts to the facemask of football helmets have resulted in larger measurement errors when compared to impacts to the shell of the helmet.^16,25,81^ Furthermore, multiple samples of a particular wearable device should be tested to evaluate inter-device reproducibility, and established standards should be followed to evaluate the repeatability of the test conditions and surrogate.^36^

### Specific Considerations

Various impact test setups are available for generating the head kinematics needed to evaluate a wearable device (Fig. 2). The drop tower is a commonly used test setup in which a headform is dropped onto a steel anvil using a monorail, twin-wire, or free-fall system.^83,99^ For example, the NOCSAE drop tower has a 1.83 m (6 ft) free fall requirement, which allows a maximum impact velocity of up to approximately 6.0 m s^−1^.^58^ Traditionally, drop tests have been focused on generating and assessing linear head kinematics; however, some setups have been modified with an angled or curved anvil to generate angular kinematics.^10,18^ While some studies have used a headform with a rigid neck,^30^ others have used a flexible neck and have simulated effective torso mass.^13^

Impactors are also commonly used to evaluate wearable devices in the laboratory and are capable of generating a broad range of 6DOF head kinematics.^5,42,53,81,90^ Impactor tests involve a ram powered by gravity (pendulum) or pneumatic/spring actuators (linear impactor) to impact a test surrogate that is typically mounted to an adjustable slider table with a flexible neck.^5^ The slider table enables simulation of torso displacement post-impact and allows the user to adjust the direction and location of the impact surface relative to the test surrogate.^5^ For example, pneumatic linear impactors have been designed to simulate high speed impacts based on video analysis of head impacts in American football.^5,30,91^ While drop tests are highly repeatable and usually cost less, impactor tests have the ability to reproduce more complex 6DOF kinematics, provide higher input energy, and simulate impacting surfaces with various stiffness and mass.

Along with the test setup, test conditions should be chosen based on the anticipated on-field impact conditions. For example, helmeted sports and non-helmeted sports differ in impact magnitude, duration, and direction, which need to be accounted for in test conditions.^42,68,69,93^ Activity- or sport-specific considerations may also be needed to account for variations in design of protective headgear^1^ and on-field impact characteristics. Centric and non-centric impact locations should be included in test conditions,^90^ while further on-field analysis should be used to identify the most common and severe test conditions.^5^ In addition, the mechanics of the impacting surface can affect impact kinematics. For example, helmet fit is not only a factor in the accuracy of a helmet-mounted device, but could also affect headform kinematics.^11^ Impacts involving a rotational component acting on a helmet-mounted device are affected by the friction between the headform scalp and helmet liner. The static and dynamic coefficients of friction for cadaver scalps, both with and without hair (mean ± SD, 0.29 ± 0.07), were significantly lower (*p* < 0.001) than for the Hybrid III head (mean ± SD, 0.75 ± 0.06).^89^ Some studies have used nylon skullcaps on the Hybrid III head to reduce friction between the head and helmet,^8,71,91^ and a different study demonstrated a 10% reduction in peak resultant kinematics in side and eccentric impacts when simulating hair and a looser fitting helmet.^1,11^ Temperature and differing recovery intervals have also been shown to affect the response of deformable elements incorporated into the impactor surface. Standards on proper measurement practices are applicable to control these effects.^4,61^

Because substantial error in the kinematics can result from insufficient coupling between the wearable device and the skull,^45,81,94^ test conditions should also be selected to evaluate factors that could contribute to coupling errors. Examples include helmet fit and facemask/chinstrap interactions affecting helmet-mounted devices, jaw mechanics affecting instrumented mouthguards, and skin/hair mechanics affecting devices mounted on the skin or headgear.^89,98^ A tighter fitting helmet has been shown to improve the accuracy of kinematic measurements from a helmet-mounted device.^38^ However, comfortable helmet fit is prioritized by athletes, and in-laboratory testing should not use unrealistically tight helmet fit to improve the accuracy of the kinematics. Validation testing of instrumented mouthguards have shown that clenching of the jaw can also substantially improve kinematic accuracy by improving the coupling of the mouthguard to the skull.^45^ However, it is possible that loose jaw conditions may occur on-field which would affect the accuracy of an instrumented mouthguard.

## Data Collection

The overall goal of data collection is to acquire laboratory-grade reference sensor data, which can then be used to quantify the errors and uncertainties present in the data from the wearable device.

### Key Considerations

Given the objective is to determine the accuracy of head impact measurements, ground truth (reference) data together with data from the wearable device should be collected for comparison. Therefore, the type and arrangement of the reference sensor is a key consideration. Mounting to the surrogate and the maximum severity of the test conditions are also important considerations when choosing a reference sensor. Another key consideration is the selection of data acquisition parameters, which are responsible for sampling head impact data. After the data are collected, raw measurements made by the wearable device and reference sensor must be post-processed to enable meaningful comparisons. Thus, the methods to post-process raw data are also important to consider. These considerations should be weighed against the study objectives and intended application. For example, study objectives such as validating impact counts have different requirements for data collection than those focused on validating measurements derived from kinematics.

### Recommended Approach

Consider the requirements for ground truth measurements in the target application, then select reference sensor arrangement. Applications that require 6DOF kinematics should choose reference arrangements capable of making these measurements.^39,62^ Applications that focus on impact counting or measuring impact direction may require only linear acceleration or high-speed video for validation.^94^ Select a laboratory-grade reference sensor that is capable of measuring the range of kinematics expected in the conditions tested. The wearable device should be mounted on the test surrogate similar to how it will be worn on-field, whereas the reference sensor should be rigidly mounted to the surrogate such that the relative location and orientation of the sensors in the wearable device and anatomical coordinate system are known.^69^

Once reference sensor and mounting have been determined, parameters for acquiring data should be set up to enable collection of independent and unbiased data for comparison. To provide complete reference information for comparison, reference sensor measurement range, sampling frequency, and recording duration should exceed those used for the wearable device. For example, reference sensors used in laboratory tests involving head impacts have sample rates on the order of 10 kHz, such that filtered reference data can be compared with wearable device data sampled on the order of 1 kHz.^76,81,93^ For applications using video, ensure that frame rates are adequate (e.g., a minimum of 1000 frames per second has been used in laboratory studies to track velocity and displacement) and that the time of impact on video can be reliably synced with data from the wearable device.^94^

**Figure 3:**
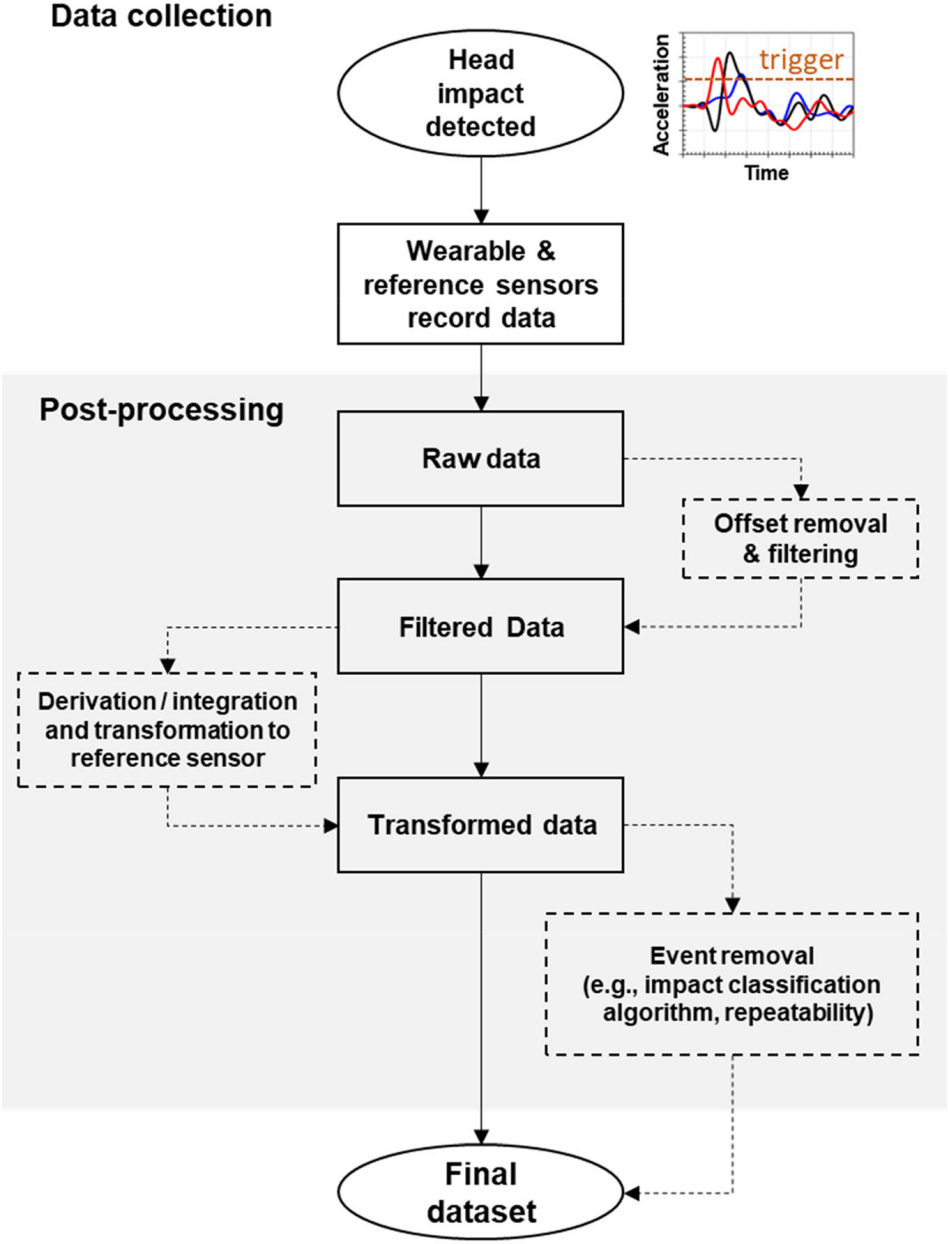
Data collection and post-processing flowchart for head impact measurement. Head impact detected constitutes 6DOF kinematics measured by the wearable device. Solid boxes indicate the result of a processing step shown in the dashed boxes

Raw data from the wearable device and reference sensor should be post-processed for comparison (Fig. 3). Head kinematics should be filtered to remove spurious frequencies. When designing a filter, consider the mechanics of the surrogate under the conditions tested, and any bandwidth requirements so that the filter maximizes the removal of noise while preserving the impact signal.^93^ Validation studies typically use Appendix C of the Society of Automotive Engineers (SAE) J211 protocol to design filters with specified channel frequency class (CFC) magnitudes.^37^ Finally, calculate any kinematic parameters that are required to transform measurements to a common point and reference frame.^37,45^

### Specific Considerations

Reference sensor arrangements should be considered in terms of their cost, mounting constraints for the surrogate, and kinematics measured by the wearable device (Table 2). Some reference sensor arrangements consist of an array of linear accelerometers [e.g., the nine-accelerometer array package (NAP)],^62^ whereas others include angular rate sensors (e.g., 3*a*3ω and 6*a*3ω).^40^ The 3*a*3ω has lower cost due to the use of fewer sensors and is relatively easy to mount to a surrogate; however, the derivative is used to approximate angular acceleration, which can amplify noise under higher frequency excitation, which should be considered when validating wearable devices that measure angular acceleration.^14,25,39,57^ While angular acceleration is algebraically calculated using the NAP and 6*a*3ω,^40^ integration is used to approximate angular velocity from the NAP, which can manifest error due to signal drift.^14,75^ The advantage of the 6*a*3ω over the NAP is that it enables direct measurement of angular velocity, which is important for validating wearable devices that measure angular rate. Furthermore, methods that leverage sensor redundancy to ensure consistency with rigid body motion should be used to check for potential sources of measurement error in reference sensor arrangements (NAP and 6*a*3ω).^75,87^

**Table 2:** Examples of different reference sensor arrangements.

Arrangement	Number of sensors	Direct measurements	Indirect measurements	Sensor redundancy
3–2–2–2 (NAP)	9	Linear acceleration, angular acceleration	Angular velocity	Yes
3a3ω	6	Linear acceleration, angular velocity	Angular acceleration	No
6a3ω	9	Linear acceleration, angular acceleration, angular velocity	–	Yes

The choice of sensor model for the reference setup should prioritize higher cost, laboratory-grade (accuracy and bandwidth), and robust sensors designed and rigorously tested for high intensity shock testing, such as piezoelectric and Integrated Electronics Piezo-Electric (IEPE) accelerometers and high-performance angular rate sensors. These sensors may present 3–4 orders of magnitude higher cost than miniature low-cost MEMS sensor alternatives used in wearable devices, but they provide high-range, high sample rate and bandwidth, low-noise measurements with individual sensor calibration and noise performance information.

When selecting reference sensor arrangements for specific surrogates, consider the constraints for mounting. For example, arrangements for the NAP and 6*a*3ω should consider the space available for mounting because the accuracy of angular acceleration calculated from an array of linear accelerometers depends on the distance between co-planar sensors.^62,87^ For example, while the Hybrid III can accommodate a range of reference sensors due to the availability of space inside the head, it may be difficult to mount similar arrangements in surrogates that have less space (e.g., the NOCSAE headform).^22,33^ When testing PMHS, choose a mounting location for the reference sensor that is away from the impact site and significant soft tissue, as these factors can reduce coupling.^73,93,97^ Obtaining reference data from volunteers is limited by the availability of mounting options; however, several studies have used biteplates and validated instrumented mouthpieces to obtain reference measurements, whereas others have used high-speed video to obtain head displacement information.^94^

When choosing data acquisition parameters, consider the duration and minimum severity of the test conditions. Wearable devices typically use a threshold based on linear acceleration to trigger data collection (e.g., 10–15 g);^21^ however, use of a trigger based on angular kinematics may also be needed in some conditions, because linear acceleration varies across a rigid body during 6DOF motion.^81,92^ When selecting parameters for the recording duration, ensure that sufficient data are collected to accomplish the study objectives. For example, duration requirements for head impact counting are typically shorter than those for kinematic measurements, whereas requirements for measuring acceleration magnitudes are shorter than those for measuring the complete time-history needed to obtain velocity and displacement and tissue-level response from Finite Element (FE) models of the head.^48^

When post-processing raw data, consider the output of the wearable device and the steps required to enable comparison with reference sensor data. In some cases, data are provided in a processed format that can be readily used for analysis, but in others, data are provided in raw format and must be post-processed.^81^ Sensor offset in the vertical axis should be removed prior to filtering in test conditions involving an initially stationary surrogate. In conditions involving a nonstationary surrogate, sensor offset should be quantified separately from pre-impact motion. Kinematic data are usually transformed to the local head coordinate system at head CG with sensor axes aligned to anatomical directions; however, some studies have examined data at other locations (e.g., to check coupling between the skull and jaw or to isolate linear and rotational sensor errors).^25,45^

Beyond standard exclusion criteria for removing tests with corrupt data or those falling outside pre-established repeatability standards,^81^ head impact classification algorithms may be used to remove non-impact recordings made by the wearable device.^65^ Clear and consistent methods for event removal criteria should be reported,^81^ and laboratory performance of the algorithm in head impact counting should be evaluated and reported prior to on-field testing. Impact classification algorithms that remove moderate or severe laboratory impacts should be noted and reported, because their removal may lead to incomplete representation of the accuracy of the wearable device.

## Data Analysis

The overall goal of the data analysis is to extract the most appropriate validation metrics and statistics to compare between the wearable device and reference sensor based on the intended application.

### Key Considerations

Processed data from the wearable device should be compared with reference sensor data using methods that enable a meaningful and unbiased assessment of accuracy. Therefore, the target application and the type of data provided by the device are key considerations. Within these considerations, the choice of validation metrics and statistical methods used to treat the data are important to consider. For example, the methods used to validate the ability of the wearable device to count head impacts differ from those used to validate the accuracy of device’s kinematic measurements. Furthermore, different methods should be considered for validating the magnitude, direction, and the time-history of head kinematics.

### Recommended Approach

For accurate head impact counting, the wearable device must consistently record impacts while minimizing recordings of spurious non-impact events. Distinguishing between head impacts and spurious events is a binary classification problem, thus binary classification metrics like recall and precision are recommended to evaluate impact counting accuracy. Recall (sensitivity) measures the percent of actual head impacts that the device records, whereas precision or positive predictive value (PPV) measures the percent of device recordings that correspond to an actual head impact.

When assessing the accuracy of a wearable device’s kinematic measurements, consider the target application and prioritize the use of validation metrics that more closely correlate with the underlying injury mechanism. Validation metrics are used to summarize the severity of a head impact and consist of metrics that are obtained either directly from the kinematics or indirectly through simulations that involve applying the kinematics to computational models that simulate tissue-level brain response.^19,49^ Applications that involve measuring head impact severity should prioritize the use of validation metrics that incorporate the magnitude, direction, and duration of 6DOF kinematics given their correlation with head injury mechanisms.^28,34,84^

Select statistical methods that provide an unbiased evaluation of the accuracy and uncertainty of a wearable device’s measurements. While simple linear regression can be used to establish the strength of the correlation between measurements from wearable device and reference sensor, the error should be quantified.^7,16,25,49,81^ Device performance should also be evaluated in different impact conditions (e.g., speeds and locations), because analysis of overall kinematic accuracy may hide egregious errors that can occur in certain conditions.^81^

### Specific Considerations

When selecting binary metrics for assessing head impact counting accuracy, consider the different sources of error. Precision and recall can be used to assess the functionality and recording threshold (trigger) of a wearable device in the laboratory (e.g., the device did not record an impact (false negative) or the device recorded data absent of head impact (false positive)). These metrics can also be used to assess the performance of an impact classification algorithm that will eventually be used with on-field data.^21^ These assessments may also clarify the source of error (e.g., the device did not record data during an impact versus a post-processing algorithm misclassified a recording as a non-impact), because it may be possible to use the data recorded by a device from laboratory tests to improve the performance of an impact classifier for future on-field use. While laboratory validation of impact counting can help to identify errors in the functionality of the device and optimize trigger thresholding and head impact classification algorithms, further quantification of false positives and false negatives through on-field validation of head impacts is recommended.

When validating kinematic accuracy, the maximum values of the resultant time histories of linear and angular acceleration and velocity (peak kinematics) are the most basic aggregate metrics for assessing impact severity.^52^ Thus, comparing peak kinematics measurements from the wearable device and reference sensor constitutes a minimum level of validation. The change in velocity (linear and angular) should be prioritized over peak resultant velocity given that the initial conditions for velocity on-field may not be known. The change in angular velocity also has higher correlation with maximum brain strain than peak resultant angular velocity for head impacts that are shorter in duration than the natural period of the brain-skull system.^24,26,43,51^ It should be noted that a weakness of relying on peak resultant values is that they can hide large changes in the directions of the applied accelerations and velocity changes that occur during an impact.

The direction of head motion during impact is an important factor for injury tolerance.^85,86^ Measures of head impact direction include metrics based on kinematic components that summarize the primary axis or plane of head motion. Incorporating direction into validation metrics is the next important step of kinematic validation. Some wearable devices estimate the location of head impact using the direction of head kinematics^46^ or through regional categorization of spherical coordinate vectors, in which accuracy can be evaluated by precision and recall and spherical methods.^47,81^ When accounting for impact direction, on-field data suggest that athletes are more susceptible to injury from temporal impacts,^54^ and FE models suggest that brain injury tolerance to rotation in the horizontal plane may be lower (up to 50%) than the coronal or sagittal plane.^86^ Furthermore, biomechanics metrics that incorporate direction of head kinematics improve prediction of tissue-level metrics based on brain strain.^24,86^

While metrics based on the peak resultant and/or directional components of head kinematics offer easy means to summarize the severity of a head impact, they are based on a single or several points from the time-history. Incorporating more information from the time-history of head kinematics improves prediction of injury by considering the magnitude, duration, shape, and direction of the pulse.^84,96^ Therefore, the highest level of kinematic validation evaluates the complete time-history of component linear and angular head kinematics. This level of validation is recommended because it enables the calculation of head injury criteria and tissue-level response from FE models from head kinematics measured by wearable devices.^48^

For validation of impact counting as a binary classification problem, studies have reported sensitivity, specificity, accuracy, and precision as common summary statistics.^95^ Linear regression of peak kinematics has been commonly applied to determine the agreement between device and reference measurements, where the slope, intercept, and the coefficient of determination (*R*^2^) are usually reported to quantify the strength of the correlation.^16,81^ Impact directions are often reported in spherical coordinate systems with error statistics such as the standard deviation ellipse (SDE).^81^ Some methods that have been used to compare time-history kinematics include, for example, the normalized root-mean-square error (NRMSE) and cross-correlation methods from the analysis software, CORrelation and Analysis (CORA).^16,27^ Statistical treatment of the data should be assessed using data aggregated across all impact conditions, as well as within individual test conditions (e.g., for each impact location).

## Discussion

Wearable devices that are validated enable collection of reliable head impact data. Although there have been numerous laboratory validation studies of wearable devices, the methods vary with no standardized approach. This paper presents recommended best practices for appropriate in-laboratory validation of the accuracy of wearable head kinematic devices. Key considerations were identified for four aspects of validation including the selection of test surrogates, test conditions, data collection, and data analysis methods. Recommendations for in-laboratory validation methods were developed based on the key considerations, and specific considerations were used to provide examples of additional crucial factors in validation as well as studies that help illustrate specific approaches.

Laboratory testing provides a necessary first step in the validation of a wearable device; however, a properly executed laboratory validation study does not ensure that the device is perfect or valid for on-field use. Thus, further validation of device-specific factors influencing on-field accuracy (e.g., head to wearable device coupling) through additional in-laboratory or on-field testing is highly recommended prior to wide-scale use. Laboratory testing is limited to examining factors that influence sensor accuracy to the extent that they are reliably recreated in the laboratory. Factors that influence coupling and fit including hair, sweat, and saliva for example, can be recreated in the laboratory to some degree; however, it is difficult to quantify the effect of these factors on the accuracy of kinematics measured on-field. For example, sweat may change the friction between the helmet and head or reduce the adhesion of a skin patch. Saliva or motion of the mouthguard relative to the teeth (repeated removal and insertion) may create conditions with poor or deteriorating sensor coupling overtime. Validation of on-field kinematics is further complicated by the lack of reliable reference measurements with which to assess the accuracy of the wearable device. As such, users and reviewers should be aware of the potential limitations in on-field device accuracy, even when a device has been validated in laboratory testing.

The current study focuses on validation of wearable device accuracy under HAE that result from direct blows to the head (impacts). HAE due to indirect or inertial loading through the neck may have relatively low magnitude, high duration kinematics compared to head impacts, and they would impose different validation requirements.^67,94^ Furthermore, aspects of device validation covered in this paper are focused primarily on use of validation metrics associated with brain injury. Other injury mechanisms (e.g., skull fracture) may require additional kinematic validation due to the higher magnitude and bandwidth requirements on linear kinematics.^94^

To-date, most laboratory validation studies have used 50th percentile adult, male ATD headforms; however, we recommend that headforms be selected based on the population involved in the intended application. Developers of a particular wearable device typically make assumptions about the user. For example, helmets assume proper fit, skin patches assume tight coupling with the skull through the skin/tissue, and all devices assume head geometry to determine the orientation with respect to the anatomical planes of the head and location of the head CG. These factors vary, on average, between males and females, across age, and ethnicity.

In summary, we suggest that the best practices recommendations outlined in this paper be referred to by developers and users of wearable head kinematic devices, as well as anyone reviewing such research. Standardized laboratory validation of wearable devices is a required step towards the collection of dependable on-field human head impact data for head injury research. Wearable devices also have potential utility in exposure monitoring and injury screening, and proper validation will continue to be critical to ensure their reliability in future consumer and clinical applications.

## References

[1] Allison MA, Kang YS, Bolte JH, Maltese MR, Arbogast KB (2014). Validation of a helmet-based system to measure head impact biomechanics in ice hockey. Med. Sci. Sports Exerc..

[2] Allison MA, Kang YS, Maltese MR, Bolte JH, Arbogast KB (2015). Measurement of hybrid III head impact kinematics using an accelerometer and gyroscope system in ice hockey helmets. Ann. Biomed. Eng..

[3] Arbogast K, Caccese J, Buckley T, McIntosh A, Henderson K, Stemper B, Solomon G, Broglio S, Funk J (2022). Consensus head acceleration measurement practices (CHAMP): origins, methods transparency and disclosure. Ann. Biomed. Eng..

[4] ASTM. Standard Practice for Conducting an Interlaboratory Study to Determine the Precision of a Test Method. 1999.

[5] Bailey AM, Sanchez EJ, Park G, Gabler LF, Funk JR, Crandall JR, Wonnacott M, Withnall C, Myers BS, Arbogast KB (2020). Development and evaluation of a test method for assessing the performance of American Football Helmets. Ann. Biomed. Eng..

[6] Bartol, A., V. Hazen, J. Kowalski, and B. Murphy. Advanced Dynamic Anthropomorphic Manikin (ADAM) Final Design Report. *astinet.dtic.mil*, 1990. https://apps.dtic.mil/sti/citations/ADA234761

[7] Bartsch A, Samorezov S, Benzel E, Miele V, Brett D (2014). Validation of an “Intelligent Mouthguard” single event head impact dosimeter. Stapp Car Crash J..

[8] Beckwith JG, Greenwald RM, Chu JJ (2012). Measuring head kinematics in football: correlation between the head impact telemetry system and hybrid III headform. Ann. Biomed. Eng..

[9] Begonia MT, Pintar FA, Yoganandan N (2018). Comparison of NOCSAE head kinematics using the hybrid III and EuroSID-2 necks. J. Biomech..

[10] Bland ML, McNally C, Zuby DS, Mueller BC, Rowson S (2020). Development of the STAR evaluation system for assessing bicycle helmet protective performance. Ann. Biomed. Eng..

[11] Bonin SJ, DeMarco AL, Siegmund GP (2018). The effect of hair and football helmet fit on headform kinematics. IRCOBI Conf..

[12] Bortenschlager, K., M. Hartlieb, A. Hirth, D. Kramberger, and S. Stahlschmidt. Detailed analysis of biorid-II response variations in hardware and simulation. https://www-esv.nhtsa.dot.gov/Proceedings/21/09-0492.pdf

[13] Bottlang M, Rouhier A, Tsai S, Gregoire J, Madey SM (2020). Impact performance comparison of advanced bicycle helmets with dedicated rotation-damping systems. Ann. Biomed. Eng..

[14] Bussone WR, Bove R, Thomas R, Richards D, Prange M (2010). Six-degree-of-freedom accelerations: linear arrays compared with angular rate sensors. SAE Tech. Pap..

[15] Byrnes K, Abramczyk J, Berliner J, Irwin A, Jensen J, Kowsika M, Mertz HJ, Rouhana SW, Scherer R, Shi Y, Sutterfield A, Xu L, Tylko S, Dalmotas D (2002). ES-2 dummy biomechanical responses. Stapp Car Crash J..

[16] Camarillo DB, Shull PB, Mattson J, Shultz R, Garza D (2013). An instrumented mouthguard for measuring linear and angular head impact kinematics in American football. Ann. Biomed. Eng..

[17] Craig MJ, Viano DC, Bir CA (2009). Jaw loading response of current ATDs. SAE Int. J. Passeng. Cars.

[18] Deck, C., N. Bourdet, F. Meyer, R. W.-J. of safety Research, and U. 2019. Protection performance of bicycle helmets. *Elsevier*, 2019. https://www.sciencedirect.com/science/article/pii/S0022437519306176 10.1016/j.jsr.2019.09.003 31862046

[19] Deck C, Willinger R (2008). Improved head injury criteria based on head FE model. Int. J. Crashworthiness.

[20] Duma SM, Manoogian SJ, Bussone WR, Brolinson PG, Goforth MW, Donnenwerth JJ, Greenwald RM, Chu JJ, Crisco JJ (2005). Analysis of real-time head accelerations in collegiate football players. Clin. J. Sport Med..

[21] Le Flao E, Siegmund GP, Borotkanics R (2022). Head impact research using inertial sensors in sport: A systematic review of methods, demographics, and factors contributing to exposure. Sports Med.

[22] Foster, J., and J. Kortge. Hybrid III—A biomechanically-based crash test dummy. *JSTOR*, 1977. https://www.jstor.org/stable/44644622

[23] Funk JR, Jadischke R, Bailey A, Crandall J, McCarthy J, Arbogast K, Myers B (2020). Laboratory reconstructions of concussive helmet-to-helmet impacts in the National Football League. Ann. Biomed. Eng..

[24] Gabler LF, Crandall JR, Panzer MB (2018). Development of a metric for predicting brain strain responses using head kinematics. Ann. Biomed. Eng..

[25] Gabler LF, Dau NZ, Park G, Miles A, Arbogast KB, Crandall JR (2021). Development of a low-power instrumented mouthpiece for directly measuring head acceleration in American Football. Ann. Biomed. Eng..

[26] Gabler LF, Joodaki H, Crandall JR, Panzer MB (2018). Development of a Single-Degree-of-Freedom Mechanical Model for Predicting Strain-Based Brain Injury Responses. J. Biomech. Eng..

[27] Gehre, C., H. Gades, and P. Wernicke. Objective rating of signals using test and simulation responses. 2009. https://www-esv.nhtsa.dot.gov/Proceedings/21/09-0407.pdf

[28] Gennarelli TA, Thibault LE, Adams JH, Graham DI, Thompson CJ, Marcincin RP (1982). Diffuse axonal injury and traumatic coma in the primate. Ann. Neurol..

[29] Goldsack JC, Coravos A, Bakker JP, Bent B, Dowling AV, Fitzer-Attas C, Godfrey A, Godino JG, Gujar N, Izmailova E, Manta C, Peterson B, Vandendriessche B, Wood WA, Wang KW, Dunn J (2020). Verification, analytical validation, and clinical validation (V3): the foundation of determining fit-for-purpose for Biometric Monitoring Technologies (BioMeTs). npj Digit. Med..

[30] Gwin JT, Chu JJ, Diamond SG, Halstead PD, Crisco JJ, Greenwald RM (2010). An investigation of the NOCSAE linear impactor test method based on *In vivo* measures of head impact acceleration in American football. J. Biomech. Eng..

[31] Hernandez F, Wu LC, Yip MC, Laksari K, Hoffman AR, Lopez JR, Grant GA, Kleiven S, Camarillo DB (2015). Six degree-of-freedom measurements of human mild traumatic brain injury. Ann. Biomed. Eng..

[32] Higgins M, Halstead PD, Snyder-Mackler L, Barlow D (2007). Measurement of impact acceleration: mouthpiece accelerometer versus helmet accelerometer. J. Athl. Train..

[33] Hodgson VR (1975). National Operating Committee on Standards for Athletic Equipment football helmet certification program. Med. Sci. Sports.

[34] Holbourn AHS (1943). Mechanics of head injuries. Lancet.

[35] Huber CM, Patton DA, Wofford KL, Margulies SS, Cullen DK, Arbogast KB (2021). Laboratory assessment of a headband-mounted sensor for measurement of head impact rotational kinematics. J. Biomech. Eng..

[36] ISO 15830-1:2013(E) Road Vehicles—Design and Performance Specifications for the WorldSID 50th Percentile Male Side Impact Dummy—Part 1: Terminology and Rationale. 2nd ed. Geneva: International Standards Organization, 2013.

[37] J211/1: Instrumentation for Impact Test - Part 1 - Electronic Instrumentation - SAE Internationalat https://www.sae.org/standards/content/j211/1_201403/

[38] Jadischke R, Viano DC, Dau N, King AI, McCarthy J (2013). On the accuracy of the Head Impact Telemetry (HIT) System used in football helmets. J. Biomech..

[39] Kang YS, Goldman S, Moorhouse K, Bolte J (2017). Evaluation of a coplanar 6a3ω configuration in the Hybrid III 50th percentile male head. Traffic Inj. Prev..

[40] Kang YS, Moorhouse K, Bolte JH (2011). Measurement of Six degrees of freedom head kinematics in impact conditions employing six accelerometers and three angular rate sensors (6aω configuration). J. Biomech. Eng..

[41] Kendall M, Walsh ES, Hoshizaki TB (2012). Comparison Between Hybrid III and Hodgson-WSU Headforms by Linear and Angular Dynamic Impact Response.

[42] Kieffer EE, Begonia MT, Tyson AM, Rowson S (2020). A two-phased approach to quantifying head impact sensor accuracy: in-laboratory and on-field assessments. Ann. Biomed. Eng..

[43] Kleiven S (2006). Evaluation of head injury criteria using a finite element model validated against experiments on localized brain motion, intracerebral acceleration, and intracranial pressure. Int. J. Crashworthiness.

[44] Kuo C, Patton D, Rooks T, Tierney G, McIntosh A, Lynall R, Esquivel A, Daniel R, Kaminski T, Mihalik J, Dau N, Urban J (2022). Consensus head acceleration measurement practices (CHAMP)—on-field deployment and validation for wearable devices. Ann. Biomed. Eng..

[45] Kuo C, Wu LC, Hammoor BT, Luck JF, Cutcliffe HC, Lynall RC, Kait JR, Campbell KR, Mihalik JP, Bass CR, Camarillo DB (2016). Effect of the mandible on mouthguard measurements of head kinematics. J. Biomech..

[46] Kuo C, Wu L, Loza J, Senif D, Anderson SC, Camarillo DB (2018). Comparison of video-based and sensor-based head impact exposure. PLoS One.

[47] Leong P (1998). Methods for spherical data analysis and visualization. J. Neurosci. Methods.

[48] Liu Y, Domel AG, Cecchi NJ, Rice E, Callan AA, Raymond SJ, Zhou Z, Zhan X, Li Y, Zeineh MM, Grant GA, Camarillo DB (2021). Time window of head impact kinematics measurement for calculation of brain strain and strain rate in American Football. Ann. Biomed. Eng..

[49] Liu Y, Domel AG, Yousefsani SA, Kondic J, Grant G, Zeineh M, Camarillo DB (2020). Validation and comparison of instrumented mouthguards for measuring head kinematics and assessing brain deformation in football impacts. Ann. Biomed. Eng..

[50] MacGillivray S, Wynn G, Ogle M, Shore J, Carey JP, Dennison CR (2021). Repeatability and biofidelity of a physical surrogate neck model fit to a hybrid III head. Ann. Biomed. Eng..

[51] Margulies SS, Thibault LE (1989). An analytical model of traumatic diffuse brain injury. J. Biomech. Eng..

[52] Margulies SS, Thibault LE (1992). A proposed tolerance criterion for diffuse axonal injury in man. J. Biomech..

[53] McIntosh A (2003). Evaluation of cricket helmet performance and comparison with baseball and ice hockey helmets. Br. J. Sports Med..

[54] Mcintosh AS, Patton DA, Fréchède B, Pierré P-A, Ferry E, Barthels T (2014). The biomechanics of concussion in unhelmeted football players in Australia: A case–control study. Br. J. Sports Med..

[55] Mertz HJ (1985). Biofidelity of the hybrid III head. SAE Tech Paper.

[56] Miller LE, Kuo C, Wu LC, Urban JE, Camarillo DB, Stitzel JD (2018). Validation of a custom instrumented retainer form factor for measuring linear and angular head impact kinematics. J. Biomech. Eng..

[57] Nevins D, Smith L, Petersen P (2019). An improved method for obtaining rotational accelerations from instrumented headforms. Sport. Eng..

[58] Nocsae. Standard test method and equipment used in evaluating the performance characteristics of protective headgear/equipment NOCSAE DOC (ND) 001- 11m13 National Operating Committee. 2013.

[59] Nusholtz GS, Aoun Z, Diomenico L, Hsu T, Gracián MA, Prado JA (2013). Statistical considerations for evaluating biofidelity, repeatability, and reproducibility of ATDs. SAE Int. J. Trans. Saf..

[60] O’Connor KL, Rowson S, Duma SM, Broglio SP (2017). Head-impact-measurement devices: a systematic review. J. Athl. Train..

[61] Organization, I. S. Accuracy (trueness and precision) of measurement methods and results - Part 3 Alternative methods for the determination of the precision of a standard measurement method. 1994.

[62] Padgaonkar AJ, Krieger KW, King AI (1975). Measurement of angular acceleration of a rigid body using linear accelerometers. J. Appl. Mech..

[63] Panzer, M. B., C. R. Dale Bass, R. S. Salzar, J. Pellettiere, and B. Myers. Evaluation of ear-mounted sensors for determining impact head acceleration. 2009.

[64] Parent D, Craig M, Moorhouse K (2017). Biofidelity evaluation of the THOR and hybrid III 50th percentile male frontal impact anthropomorphic test devices. Stapp Car Crash J..

[65] Patton DA (2016). A review of instrumented equipment to investigate head impacts in sport. Appl. Bionics Biomech..

[66] Pellman EJ, Viano DC, Tucker AM, Casson IR, Waeckerle JF, Maroon JC, Lovell MR, Collins MW, Kelly DF, Valadka AB, Cantu RC, Bailes JE, Levy ML (2003). Concussion in professional football: reconstruction of game impacts and injuries. Neurosurgery.

[67] Reynier KA, Alshareef A, Sanchez EJ, Shedd DF, Walton SR, Erdman NK, Newman BT, Giudice JS, Higgins MJ, Funk JR, Broshek DK, Druzgal TJ, Resch JE, Panzer MB (2020). The effect of muscle activation on head kinematics during non-injurious head impacts in human subjects. Ann. Biomed. Eng..

[68] Reynolds BB, Patrie J, Henry EJ, Goodkin HP, Broshek DK, Wintermark M, Druzgal TJ (2017). Comparative analysis of head impact in contact and collision sports. J. Neurotrauma.

[69] Rich AM, Filben TM, Miller LE, Tomblin BT, Van Gorkom AR, Hurst MA, Barnard RT, Kohn DS, Urban JE, Stitzel JD (2019). Development, validation and pilot field deployment of a custom mouthpiece for head impact measurement. Ann. Biomed. Eng..

[70] Rowson B, Tyson A, Rowson S, Duma S (2018). Measuring head impacts: accelerometers and other sensors. Handb. Clin. Neurol..

[71] Rowson S, Beckwith JG, Chu JJ, Leonard DS, Greenwald RM, Duma SM (2011). A six degree of freedom head acceleration measurement device for use in football. J. Appl. Biomech..

[72] Rowson S, Duma SM (2013). Brain injury prediction: assessing the combined probability of concussion using linear and rotational head acceleration. Ann. Biomed. Eng..

[73] Salzar RS, Bass CRD, Pellettiere JA (2008). Improving earpiece accelerometer coupling to the head. SAE Int. J. Passeng. Cars Mech. Syst..

[74] Sances A, Kumaresan S (2001). Comparison of biomechanical head-neck responses of hybrid III dummy and whole body cadaver during inverted drops. Biomed. Sci. Instrum..

[75] Sanchez EJ, Gabler LF, Good AB, Funk JR, Crandall JR, Panzer MB (2019). A reanalysis of football impact reconstructions for head kinematics and finite element modeling. Clin. Biomech..

[76] Sandmo SB, McIntosh AS, Andersen TE, Koerte IK, Bahr R (2019). Evaluation of an in-ear sensor for quantifying head impacts in youth soccer. Am. J. Sports Med..

[77] Schmidt AL, Ortiz-Paparoni MA, Shridharani JK, Nightingale RW, Bass CR (2018). Time and temperature sensitivity of the Hybrid III neck. Traffic Inj. Prev..

[78] Shaw G, Crandall J, Butcher J (2002). Comparative evaluation of the THOR advanced frontal crash test dummy. Int. J. Crashworthiness.

[79] Siegmund, G. P., S. J. Bonin, J. F. Luck, and C. R. D. Bass. Validation of a skin-mounted sensor for measuring in-vivo head impacts. 2015. <http://www.ircobi.org/wordpress/downloads/irc15/pdf_files/28.pdf>

[80] Siegmund GP, Guskiewicz KM, Marshall SW, Demarco AL, Bonin SJ (2014). A headform for testing helmet and mouthguard sensors that measure head impact severity in football players. Ann. Biomed. Eng..

[81] Siegmund GP, Guskiewicz KM, Marshall SW, DeMarco AL, Bonin SJ (2016). Laboratory validation of two wearable sensor systems for measuring head impact severity in football players. Ann. Biomed. Eng..

[82] Siegmund, G. P., B. E. Heinrichs, J. M. Lawrence, and M. M. G. M. Philippens. Kinetic and kinematic responses of the RID2a, hybrid III and human volunteers in low-speed rear-end collisions. 2001. 10.4271/2001-22-0011 17458748

[83] Standard Z90.4 American National Standard for Protective headgear for bicyclists. New York, NY: 1984.

[84] Stapp JP (1957). Human tolerance to deceleration. Am. J. Surg..

[85] Sullivan S, Eucker SA, Gabrieli D, Bradfield C, Coats B, Maltese MR, Lee J, Smith C, Margulies SS (2015). White matter tract-oriented deformation predicts traumatic axonal brain injury and reveals rotational direction-specific vulnerabilities. Biomech. Model. Mechanobiol..

[86] Takhounts, E. G., M. J. Craig, K. Moorhouse, J. McFadden, and V. Hasija. Development of brain injury criteria (BrIC). 2013. 10.4271/2013-22-0010 24435734

[87] Takhounts EG, Hasija V, Eppinger RH (2009). Analysis of 3D rigid body motion using the nine accelerometer array and the randomly distributed in-plane accelerometer systems. ESV.

[88] Tiernan S, Byrne G, O’Sullivan DM (2019). Evaluation of skin-mounted sensor for head impact measurement. Proc. Inst. Mech. Eng. Part H.

[89] Trotta A, Ní Annaidh A, Burek RO, Pelgrims B, Ivens J (2018). Evaluation of the head-helmet sliding properties in an impact test. J. Biomech..

[90] Tyson AM, Duma SM, Rowson S (2018). Laboratory evaluation of low-cost wearable sensors for measuring head impacts in sports. J. Appl. Biomech..

[91] Viano DC, Withnall C, Halstead D (2011). Impact performance of modern football helmets. Ann. Biomed. Eng..

[92] Wang T, Kenny R, Wu LC (2021). Head impact sensor triggering bias introduced by linear acceleration thresholding. Ann. Biomed. Eng..

[93] Wu LC, Laksari K, Kuo C, Luck JF, Kleiven S, Bass CR, Camarillo DB (2016). Bandwidth and sample rate requirements for wearable head impact sensors. J. Biomech..

[94] Wu LC, Nangia V, Bui K, Hammoor B, Kurt M, Hernandez F, Kuo C, Camarillo DB (2016). *In vivo* evaluation of wearable head impact sensors. Ann. Biomed. Eng..

[95] Wu LC, Zarnescu L, Nangia V, Cam B, Camarillo DB (2014). A head impact detection system using SVM classification and proximity sensing in an instrumented mouthguard. IEEE Trans. Biomed. Eng..

[96] Wu T, Sato F, Antona-Makoshi J, Gabler LF, Giudice JS, Alshareef A, Yaguchi M, Masuda M, Margulies SS, Panzer MB (2022). Integrating human and nonhuman primate data to estimate human tolerances for traumatic brain injury. J. Biomech. Eng..

[97] Yoganandan N, Zhang J, Pintar FA, King Liu Y (2006). Lightweight low-profile nine-accelerometer package to obtain head angular accelerations in short-duration impacts. J. Biomech..

[98] Zouzias D, De Bruyne G, Ni Annaidh A, Trotta A, Ivens J (2021). The effect of the scalp on the effectiveness of bicycle helmets’ anti-rotational acceleration technologies. Traffic Inj. Prev..

[99] US Department of Transportation (DOT), National Highway Traffic Safety Administration (2003). Laboratory Test Procedure for FMVSS 218 Motorcycle Helmets. TP-218-04.

